# Tumor-suppressing potential of stingless bee propolis in *in vitro* and *in vivo* models of differentiated-type gastric adenocarcinoma

**DOI:** 10.1038/s41598-019-55465-4

**Published:** 2019-12-23

**Authors:** Mark Joseph Desamero, Shigeru Kakuta, Yulan Tang, James Kenn Chambers, Kazuyuki Uchida, Maria Amelita Estacio, Cleofas Cervancia, Yuri Kominami, Hideki Ushio, Jun Nakayama, Hiroyuki Nakayama, Shigeru Kyuwa

**Affiliations:** 1 Laboratory of Biomedical Science, Graduate School of Agricultural and Life Sciences, The University of Tokyo, 1-1-1, Yayoi, Bunkyo-ku, Tokyo, 113-8657 Japan; 2 Department of Basic Veterinary Sciences, College of Veterinary Medicine, University of the Philippines Los Baños, Laguna, 4031 Philippines; 3 UPLB Bee Program, University of the Philippines Los Baños, Laguna, 4031 Philippines; 4 Institute of Biological Sciences, College of Arts and Sciences, University of the Philippines Los Baños, Laguna, 4031 Philippines; 5 Laboratory of Veterinary Pathology, Graduate School of Agricultural and Life Sciences, The University of Tokyo, 1-1-1, Yayoi, Bunkyo-ku, Tokyo, 113-8657 Japan; 6 Laboratory of Marine Biochemistry, Graduate School of Agricultural and Life Sciences, The University of Tokyo, 1-1-1, Yayoi, Bunkyo-ku, Tokyo, 113-8657 Japan; 7 Department of Molecular Pathology, Shinshu University School of Medicine, 3-1-1 Asahi, Matsumoto, Nagano, 3908621 Japan

**Keywords:** Nutritional supplements, Gastric cancer, Experimental models of disease

## Abstract

The protective property of propolis across a wide spectrum of diseases has long been realized, yet the anti-tumor efficacy of this bioactive substance from Philippine stingless bees has remained poorly understood. Here, we showed the tumor-suppressing potential of crude ethanolic extract of Philippine stingless bee propolis (EEP) in *in vitro* models of gastric cancer highlighting the first indication of remarkable subtype specificity towards differentiated-type human gastric cancer cell lines but not the diffuse-type. Mechanistically, this involved the profound modulation of several cell cycle related gene transcripts, which correlated with the prominent cell cycle arrest at the G0/G1 phase. To reinforce our data, a unique differentiated-type gastric cancer model, *A4gnt* KO mice, together with age-matched 60 week-old C57BL/6 J mice were randomly assigned to treatment groups receiving distilled water or EEP for 30 consecutive days. EEP treatment induced significant regression of gross and histological lesions of gastric pyloric tumors that consistently corresponded with specific transcriptional regulation of cell cycle components. Also, the considerable p21 protein expression coupled with a marked reduction in rapidly dividing BrdU-labeled S-phase cells unequivocally supported our observation. Altogether, these findings support the role of Philippine stingless bee propolis as a promising adjunct treatment option in differentiated-type gastric cancer.

## Introduction

Tremendous advances in early disease diagnosis have revolutionized the mortality landscape of gastric cancer (GC) as seen in the last decades. However, according to the latest GLOBOCAN 2018 estimates, it continues to be a major disease burden affecting approximately 783,000 individuals whose gravity has been widely observed in East Asian countries like Japan, Mongolia, and Korea^1^. GC is a highly complicated disease as evidenced by its broadly distinct morphological and biological features not to mention the multitude of genetic and epigenetic perturbations reported in several genes i.e. *TP53*, *CTNNB1*, *RUNX3*, *CDX2*, *CDKN2A*, *ERBB2*, *IL10*, *TNFA*, *IL1B*, and *RHOA*^2,3^. It has been predominantly classified in accordance with the traditional histological classification by the Lauren system as well as on the basis of their molecular gene expression signatures. Generally, differentiated-type GC consists of tumor cells thrown into tubular or glandular-like structures and displays well-demarcated progression of precursor lesions whereas diffuse-type GC presents poorly cohesive cancer cells occurring singly or in clumps with relatively unknown or less identifiable precancerous stages^4,5^.

To date, surgical resection remains the cornerstone of GC therapy with curative intent but still around 40–65% of localized GC patients are refractory showing relapse in the tumor bed, anastomosis or regional lymph nodes^6,7^. To address this gap, multimodal approaches and targeted therapies have previously emerged, thus providing considerable support to surgery alone^6,8–10^. Unfortunately, all these regimens have been plagued with serious adverse events of toxicity, which significantly influence patient’s compliance eventually leading to untimely treatment withdrawal. Therefore, in view of this apparent drawback, any alternative or auxiliary treatment modalities where GC patients could profit in terms of fewer complications and better quality of life are greatly warranted.

In recent years, renewed interests in natural products have taken the spotlight of drug discovery and cancer research^11,12^. Propolis, a bee-derived substance consisting of bees’ own salivary secretion together with collected resins, exudates, and oils, is a natural healing agent that has been highly regarded owing to its low toxicity, relative safety, and pronounced biological functionality^13^. It contains a broad diversity of chemical composition that is not only remarkably influenced by season, geographical location, bee species, extraction method, and existing vegetation but also is attributable to its putative bioactivities^14,15^. In particular reference to its anti-cancer property, propolis samples from Brazil, Netherlands, Portugal, New Zealand, Korea, Taiwan, China, and Algeria have been shown to inhibit a wide spectrum of *in vitro* human cancer cell lines of lung, stomach, cervical, esophageal, brain, laryngeal, skin, and breast carcinoma^16–21^. This response was purported to mechanistically involve the induction of proline dehydrogenase/proline hydrogenase- and DNA fragmentation-initiated apoptosis; regulation of glutathione and glutathione S-transferase; inhibition of NF-κB and JNK signaling pathways; upregulation of p53, Bax, cleaved-caspase-3 and 9, and MAPK-associated proteins; and modulation of the components of the cell cycle^22–26^. However, in spite of these great deal of evidences and convincing role in carcinogenesis, the anti-tumor potential of propolis from the native species of Philippine stingless bees (*Tetragonula biroi* Friese) is largely unknown and sparingly investigated. Previously, our group was the first to describe that crude EEP from this indigenous bee species could exert potent neuroprotective activity through abrogation of the neurologic deficit and neuronal damage in a rat model of ischemic stroke^27^. In this current report, we attempted to explore whether possible anti-cancer efficacy might also be included in its repertoire of bioactivities; hence we carried out a pharmacognostic evaluation of EEP from Philippine stingless bees with specific emphasis on its tumor-suppressing potential in *in vitro* and *in vivo* models of differentiated-type gastric adenocarcinoma.

## Materials and Methods

### Cell lines

Four human gastric cancer cell lines (AGS, MKN-45, NUGC-4, MKN-74) were utilized in this study. AGS cell line was obtained from the American Type Culture Collection, Manassas, VA, USA. MKN-45 and NUGC-4 cell lines were sourced from Riken Bioresource Center Cell Bank, Tsukuba, Ibaraki, Japan whereas MKN-74 cell line was procured from JRCB Cell Bank, Osaka, Japan. These cells were grown in RPMI 1640 medium supplemented with 10% heat-inactivated fetal bovine serum, 1% Penicillin and Streptomycin and incubated at 37 °C in a humidified chamber with 5% CO_2_.

### Propolis sample extraction and preparation

Standardized and authenticated samples of propolis from Philippine stingless bees were obtained from the UPLB Bee Program Meliponary, Institute of Biological Sciences, University of the Philippines Los Baños. As detailed in our previous publication^27^ and succinctly described here, crude extraction was performed by dissolving the recovered residues after rotary evaporation at 40 °C in an analytical grade ethanol to attain a final concentration of 300 mg/ml.

### Cell proliferation assay

Viability of each gastric cancer cell lines upon EEP treatment was ascertained by the MTT assay (Nacalai Tesque Inc., Kyoto, Japan) following the manufacturer’s instructions. Cells in 200 μl culture medium were seeded into a 96-well plate at an appropriate density and incubated with varying concentrations of EEP (blank, 0, 1, 10, 100, 250, 500, 1000 μg/ml) at three different time points as follows: 24, 48, and 72 hours. Absorbance readings at 570 nm were measured with an iMark microplate reader (BioRad, Tokyo, Japan). Two independent experiments with each experiment run in duplicates were undertaken. The cytotoxic drug Cisplatin, which was similarly tested, was used as a positive control.

Cell viability (%) as a function of that of the control was computed as follows: [(A_570_ Treated cells-A_570_ Blank)/(A_570_ Control cells-A_570_ Blank)*100]. Half minimal inhibitory concentration (IC_50_) was derived by graphically plotting the calculated % viable cells against its corresponding EEP concentrations and the one that generated the best fit was adopted.

### Cell cycle analysis

The distribution of cells in the sub-G1, G0/G1, S, G2/M, and multi-nuclear phases were analyzed using BD FACSVerse Flow Cytometer (BD Biosciences, San Jose, CA, USA) and BD FACSuite software. Briefly, AGS cells were seeded into a 6-well/flat-bottom microplate at an appropriate density. Following 24-hour incubation, cells were allowed to synchronize at G0 phase by serum deprivation for 24 hours and then treated with complete culture medium alone or EEP (IC_50_ at 48 h) for another 48 hours. Cells were harvested, washed with PBS twice, fixed with absolute ethanol for 30 minutes, stained with 50 μg/ml propidium iodide solution (Sigma-Aldrich, St. Louis, MO, USA) at 37 °C for 1 hour, and assessed accordingly using a total of 10,000 events. Triplicates of two independent experiments were performed.

### Apoptosis (TUNEL) assay

The potential efficacy of EEP to incite apoptosis through DNA fragmentation was discerned by the TUNEL assay using the MEBSTAIN apoptosis TUNEL kit direct protocol (Medical and Biological Laboratories Co., Ltd., Woburn, MA, USA). For *in vitro* experiment, aliquots of 200 μl suspended cells at an appropriate density were seeded into an 8-well culture slide and incubated for 48 hours with culture medium alone (control) or EEP whose concentration approximated the pre-determined IC_50_ value (at 48 hours) for each gastric cancer cell lines. DNA nick-end labeled cells were finally mounted in a mounting medium with DAPI (Vectashield, Vector Laboratories, Inc., Burlingame, CA, USA) and visualized under a confocal laser-scanning microscope (LSM510 Version 2.02; Carl Zeiss, Jena, Germany). Positive and negative control cells were additionally prepared as per manufacturer’s instructions. The number of gastric cancer cells undergoing apoptosis, expressed as % apoptotic cells, was estimated by counting the number of FITC-dUTP-labeled cells per 1000 cells and the average measurement from three independent counts was obtained. For *in vivo* study, deparaffinized tissue sections were subjected to proteinase K pre-treatment for 30 minutes at 37 °C prior to DNA nick-end labeling. Analysis of the number of cells undergoing apoptosis was determined by tallying the total number of FITC-dUTP- apoptotic cells per 100 μm area taken from at least three different areas.

### Animals

*A4gnt*, the gene encoding for the glycosyltransferase, α1, 4-*N*-acetylglucosaminyltransferase, is responsible for the biosynthesis of α1, 4-linked *N*-acetylglucosamine residues (αGlcNAc) found in the terminals of the gastric gland mucin-specific *O*-glycans in mice. Deletion of this gene has been shown to induce a complete loss of αGlcNAc expression accompanied by the progressive development of Differentiated-type gastric adenocarcinoma in a hyperplasia-dysplasia-carcinoma continuum that is strictly confined at the pyloric mucosa of the resulting mutant animals^28^. Similarly, loss of αGlcNAc in humans has been convincingly demonstrated in differentiated-type gastric cancer showing strong correlation with depth of invasion, venous invasion and staging^29^.

### Treatment groups

Sixty week-old *A4gnt* KO mice of both sexes, which displayed full-blown differentiated-type gastric adenocarcinoma and age-and sex-matched C57BL/6 J mice obtained from Nihon SLC were utilized in the present study. These animals were randomly allocated into four treatment groups as follows: (i) C57BL/6 J + distilled water (n = 7), (ii) C57BL/6 J + EEP (100 mg/kg BW, n = 7), (iii) *A4gnt* KO mice + distilled water (n = 10), and (iv) *A4gnt* KO mice + EEP (100 mg/kg BW, n = 10). Prior to administration, EEP was diluted to distilled water thereby bringing the vehicle ethanol to a negligible concentration. All treatments were administered daily via feeding tube for 30 consecutive days. Animals were housed in standard polycarbonate cages and maintained under specific pathogen-free condition with 12 h light: 12 h dark cycle. Rodent chow (CRF-1, Oriental Yeast Co., Ltd., Tokyo, Japan) and water were provided in *ad libitum* basis.

### Histopathology

Animals were injected intraperitoneally with Bromodeoxyuridine solution (BrdU; 10 mg/kg) an hour before sacrifice to allow labeling of rapidly proliferating S-phase cells^28^. The stomach tissues together with a small portion of the duodenum were harvested and divided into two for subsequent immunohistochemical and gene expression analyses. For histopathological examination, half of the stomach tissue was flattened in a tissue cassette with biopsy sponge pads, fixed in 10% buffered formaldehyde (pH 6.8) for at least 48 hours, and processed for routine H&E.

Semi-quantitative assessment of the influence of the respective treatments on gross gastric mucosal elevation was performed as defined by the following parameters: 0- healthy mucosa/none, 1- mildly, 2- moderately, and 3- markedly. Mucosal thickness of the pyloric mucosa, meanwhile, was determined using the prepared H&E sections by measuring from the base up to the highest point of a properly oriented gastric epithelium and the average measurement from at least three different areas was obtained^30^.

### Immunohistochemistry

Analysis of the expression of T cell marker, CD3, proliferation marker, BrdU, and cell cycle marker, p21 was accomplished through immunohistochemistry using primary antibodies, anti-CD3 (DakoCytomation, Glostrup, Denmark; polyclonal; ready-to-use), anti-BrdU (DakoCytomation, Glostrup, Denmark; clone Bu20a; 1:50 dilution), and anti-p21 (Abcam, Tokyo, Japan; clone ab188224; 1:200 dilution) respectively, as previously described^30^.

Evaluation of CD3, BrdU and p21 labeling was done by counting the number of CD3-and BrdU-positive cells in a defined 100 μm area having the highest cell density and the average count from at least three measurements was attained^30^.

### Quantitative real-time PCR (qRT-PCR)

Gene expression profiles of human gastric cancer cell lines as well as the harvested mice stomach tissues were achieved using StepOnePlus Real-Time PCR (Applied Biosystems, CA, USA). For the former, cells at suitable density were seeded into a 6-well/flat-bottom microplate containing 2 ml culture medium and incubated for 48 hours with culture medium alone (control) or EEP with concentration corresponding to the calculated IC_50_ value (at 48 hours) for each respective gastric cancer cell lines. This was performed in three independent experiments. For the latter, stomach specimens (n = 5) kept in RNA*later* solution (Ambion, St. Austin, TX, USA) were homogenized in a Shake Master Auto BMS-A20TPver.2.0 (BMS, Tokyo, Japan). RNA was extracted in all samples using Nucleospin RNA isolation kit (Macherel-Nagel, Düren, Germany) according to the manufacturer’s instructions. Synthesis of the first stand cDNA, on the other hand, was carried out using PrimeScript RT reagent kit (Perfect Real Time) (Takara Bio, Shiga, Japan).

Real Time PCR analysis was performed following the TB Green *Premix Ex Taq* II (Tli RNaseH Plus) (TakaRa Bio, Shiga, Japan) protocol and the expression levels of the succeeding genes were quantified: (Mice) *Actb, Il10, Il11, Il1b, Tnfa, Ifng, Il6, Bcl2, Bcl2l1, Bax, Bad, Trp53, Cdkn1a, Cdkn1b, Ccnd1, Cdk1, Cdk2*; (Human gastric cancer cell lines) *ACTB*, *BCL2*, *BCL2L1*, *BAX*, *BAD*, *TP53*, *CDKN1A*, *CDKN1B, CCND1, CDK1, CDK2*. The primers used for these genes are listed in Supplementary Table 1. The resulting mRNA expression was normalized to the expression of the internal control gene, *Actb* and the comparative CT value was determined by setting the average mRNA expression level of wildtype control C57BL/6 J mice given distilled water or untreated human cancer cell lines as 1.0. Analysis was run in duplicates.

### GC-MS/MS assay

The trimethylsilyl (TMS) ether derivatives of EEP were subjected to GC-MS/MS analysis. Briefly, freeze-dried EEP was oxymated with 20 mg/ml methoxyamine hydrochloride in pyridine at 30 °C for 90 minutes and silylated with MSTFA at 30 °C for 30 minutes. Derivatized EEP was then injected in a splitless mode into a GCMS-TQ8040 (Shimadzu, Kyoto, Japan) equipped with an Agilent J&W DB-5 (30 m × 0.25 mm ID, 1.00 μm film, Agilent Technologies Japan, Ltd., Tokyo, Japan). Helium was utilized as a carrier gas using a linear velocity of 39 cm/s at a constant flow rate of 1.1 ml/min. The pressure, total flow, and injection temperature were preferentially set at 83.7 kPa, 17.1 ml/min, and 280 °C, respectively. The oven program, which started with an initial temperature of 100 °C for 4 minutes, was heated up to 320 °C at a rate of 10 °C /min, and held for 11 min. The ion source was set at 200 °C whereas the interface was set at 280 °C. All spectra were recorded in the mass range of 45–600 *m/z*. Identification of detected compounds was accomplished by analyzing both chromatograms and mass spectra using GCMS solution Ver.4.45 (Shimadzu, Kyoto, Japan) and comparing to those listed in NIST 17 mass spectral library.

### Statistical analysis

All values were expressed as mean ± SD. Multiple comparisons were performed using One-Way Analysis of Variance (ANOVA) with Tukey-HSD posttest or the non-parametric Kruskal-Wallis test whereas Independent-Sample T-test or Mann-Whitney U Test was used in the case of only two treatment groups. All analyses were performed using SPSS v.23 (IBM Corp., Armonk, NY, USA) and values with *P* < 0.05 were considered statistically significant.

### Ethical clearance

All experimental animal procedures herein performed were in accordance with the guidelines and approval of the Institutional Animal Care and Use Committee, Graduate School of Agricultural and Life Sciences, The University of Tokyo (Approval No. P17–005H03).

## Results

### EEP selectively impedes proliferation of differentiated-type GC cell lines

The effect of EEP exposure on cancer cell proliferation was first screened using four *in vitro* cell lines of human GC namely, AGS, NUGC-4, MKN-45, and MKN-74. In accordance to the intrinsic genomic classification system following the two-marker method (LGALS4/CDH17) devised by Tan and colleagues^31^, the former three GC cell lines and MKN-74 were respectively categorized into differentiated-type and diffuse-type as confirmed by gene expression analysis (Supplementary Fig. 1). Dose- and time-dependent response to EEP treatment was then evaluated by incubating cells into increasing range of EEP concentration (1–1000 μg/ml) over time schedules of 24, 48, and 72 hours. As seen in Table 1, differentiated-type GC cell lines were more sensitized to the action of EEP showing a commensurate reduction in cancer cell proliferation as a function of higher concentration and longer incubation times. Notably, AGS cells exhibited the highest degree of inhibition, which at 72 hours recorded an IC_50_ value of 39 μg/ml, followed by NUGC-4 and MKN-45 with only marginally different values of 315 μg/ml and 318 μg/ml, respectively. Contrarily, MKN-74 cells appeared to be somewhat resistant as reflected by the absence of any visible decrement in the percentage of viable cells at concentrations < 500 μg/ml even after 48 hours of incubation, therefore, manifesting a greater IC_50_ value (>900 μg/ml) (Table 1).

**Table 1:** IC_50_ value of four human gastric cancer cell lines following incubation with crude EEP at different time schedules.

	IC_50_ (*μ*g/ml)			
	AGS	MKN45	NUGC4	MKN74
24 h	650	1156	580	1259
48 h	188	386	376	955
72 h	39	318	315	925
Cisplatin (48 h)	~4	~3	~2	~12

### EEP acts *in vitro* through modulation of the cell cycle and apoptotic machineries

**Figure 1:**
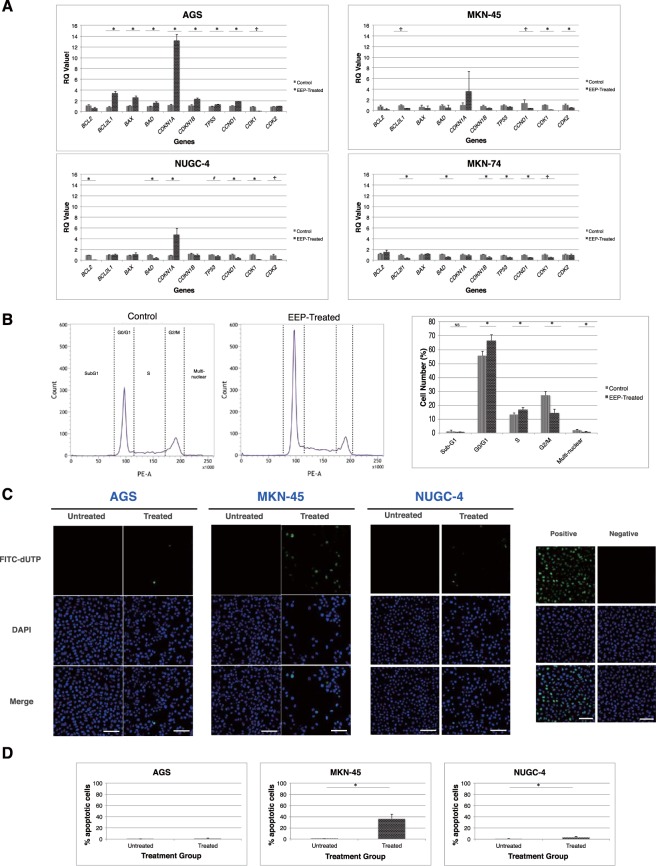
EEP acts *in vitro* through modulation of the cell cycle and apoptotic machineries. (**A**) Real time PCR profile of selected genes associated in cell cycle regulation and apoptosis in four human GC cell lines following 48 hour-incubation with either culture medium alone or EEP whose concentration approximated the determined IC_50_ value (@ 48 h) for each respective cell lines. Data shown as mean ± SD are representative of three independent experiments with each experiment run in duplicates. ^#^*P* < 0.05 and ^**^P < 0.01 using Independent sample t-test, *P* < 0.05 using Mann-Whitney U test. (**B**) Cell cycle analysis of AGS cells using propidium iodide staining depicting the representative raw data of the untreated control and EEP-treated cells (IC_50_ value @ 48 h) (left) and graphical analysis of the cell cycle distribution in the sub-G1, G0/G1, S, G2/M, and multi-nuclear phases (right). Data are shown as mean ± SD taken from triplicates of two independent experiments. NS- not significant, ^*^P < 0.01 using Independent sample t-test. (**C**) Validation of DNA fragmentation-initiated apoptosis using TUNEL assay in three sensitive human GC cell lines after application of either culture medium alone or EEP at respective IC_50_ concentration at 48 h (above). Positive control cells were subjected to DNase I treatment prior to TdT labeling while negative control cells were incubated in TdT solution in lieu of TdT reagent (below). Scale bar: 100 μm (**D**) Comparison of the number of cells undergoing apoptosis expressed as % apoptotic cells between untreated and EEP-treated groups in three human GC cell lines. ^*^P < 0.05 using Independent sample t-test.

The encouraging data of our proliferation experiment evoked further inquiries into the possible mechanisms that underpinned this perceived activity. Since the contribution of the cell cycle and apoptosis are immensely recognized in cancer^32^ and that these could be regulated by propolis as already pointed out by numerous studies^22,33,34^, the potential involvement of the present EEP sample on these processes was promptly investigated. Real time PCR analysis was performed on GC cells treated with culture medium alone (control) or EEP (IC_50_ at 48 h) to detect changes in the transcription level of selected genes implicated in cell cycle progression and apoptosis. Among these genes, a striking upregulation was characteristically disclosed by *CDKN1A* attaining a statistically significant result for AGS (*p* < 0.001) and NUGC-4 (*p* < 0.050) and a propensity for increased expression for MKN-45 (Fig. 1A, Supplementary Table 2). Noteworthily, this distinct overexpression was only established in all differentiated-type GC cell lines but not diffuse-type (MKN-74), possibly suggesting subtype-specific proclivity. In stark contrast, gene expression levels of *CDK1* and *CCND1* were significantly demoted. Also, whereas marked elevation of *CDKN1A* was accompanied by a profound increment in the levels of *CDKN1B* (*p* < 0.001) and *TP53* (*p* < 0.005) in AGS cells, *CDK2* expression levels in NUGC-4 and MKN-45 cells were concomitantly down regulated (Fig. 1A, Supplementary Table 2). On the other hand, no clear-cut pattern seemed discernible concerning the expression profile of several apoptosis-associated genes. Nevertheless, it could not be discounted that in some cancer cell lines a response suggestive of an apoptotic phenomenon was undeniably expressed such as the significant induction of *BAX* (*p* < 0.001) and *BAD* (*p* < 0.003) in AGS as well as the considerable down-expression of *BCL2L1* in MKN-45 (*p* < 0.050), and *BCL2* in NUGC4 (*p* < 0.001). In the case of MKN-74 cells, the resulting gene transcription findings firmly asserted the lack of positive response to EEP treatment (Fig. 1A, Supplementary Table 2). Moreover, cell cycle analysis and TUNEL assay were subsequently undertaken to elaborate the ability of EEP to trigger cell cycle arrest and apoptosis. As shown in Fig. 1B, EEP-treated AGS cells were significantly arrested at the G0/G1 phase showing a prominently sharpened peak, which accounts for around 66% of total cells counted as compared to only about 55% documented in the corresponding untreated cells. Additionally, this perceptible increment in G0/G1 phase following EEP treatment was accompanied by a significant augmentation in the number of S-phase cells together with a marked depletion of cells at the G2/M and multi-nuclear phases relative to those of the untreated control counterpart. No evident difference, however, was noted on sub-G1 phase cells. Meanwhile, as depicted in Fig. 1C,D, DNA fragmentation was only modestly promoted in AGS and NUGC-4 cells after EEP treatment but dramatically enhanced in MKN-45 cells wherein more than 35% of cancer cells were accounted to have undergone apoptosis.

### EEP supplementation induces promising anti-tumor response in mice model of differentiated-type gastric adenocarcinoma

**Figure 2:**
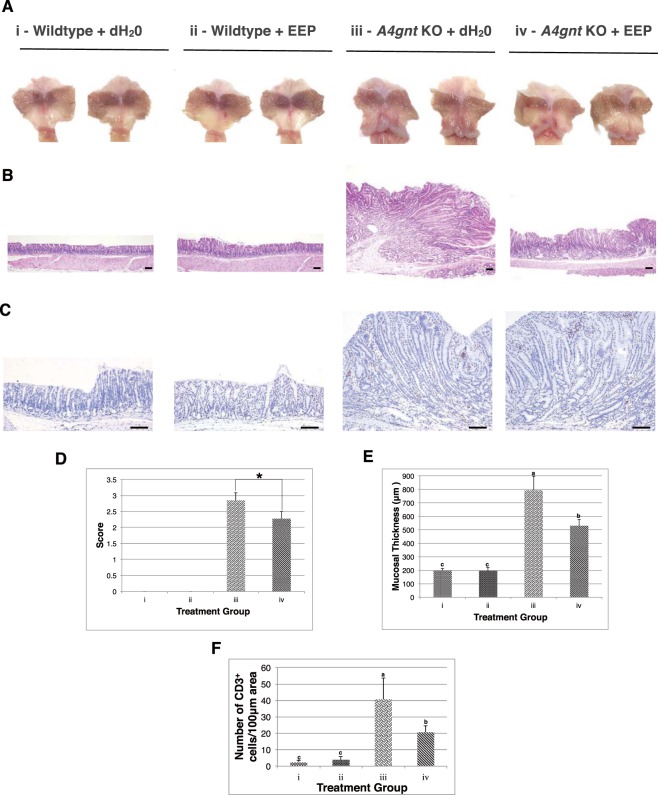
EEP supplementation induces promising anti-tumor response in mice model of differentiated-type gastric adenocarcinoma. (**A**) Representative stomach tissues of 60-week old C57BL/6 J and *A4gnt* KO mice reflecting the gross mucosal elevation of the pyloric antrum following oral administration of respective treatments for 30 consecutive days. (**B**) H&E sections of the mouse pyloric mucosa among the different treatment groups. Scale bar: 100 μm (**C**) T-lymphocyte infiltration of the pyloric mucosa among the different treatment groups as depicted by CD3 immunostaining. Scale bar: 100 μm (**D**) Comparison of the gross mucosal elevation score among the different treatment groups. 0 - none/healthy mucosa, 1 - mildly, 2 – moderately, 3 - markedly. ^*^P < 0.05 using Independent-sample t-test. (**E**) Comparison of the gastric mucosal thickness among the different treatment groups expressed as mean measurement (μm) taken from at least three different areas of the pyloric mucosa. Means with different letter are significant at *P* < 0.05 using ANOVA with Tukey-HSD posttest. (**F**) Comparison of the number of CD3-positive T-lymphocytes among the different treatment groups expressed as mean count per 100 μm area taken from at least three different points with the highest cell density. Means with different letter are significant at *P* < 0.05 using ANOVA with Tukey-HSD posttest. i – Wildtype + dH_2_0, ii – Wildtype + EEP, iii – *A4gnt* KO + dH_2_0, iv - *A4gnt* KO + EEP.

To provide a proof of concept that such *in vitro* response could be replicated in the context of a more complicated system, we considered utilizing a unique animal model that can aptly recapitulate the intricacies of differentiated-type GC. *A4gnt* KO mice deficient in the gene encoding for α1, 4-*N*-acetylglucosaminyltransferase notably develop gastric cancer in a spontaneous manner of hyperplasia-dysplasia-adenocarcinoma sequence^28^. In the present study, 60 week-old *A4gnt* KO mice exhibiting full-blown gastric adenocarcinoma, and matched wildtype C57BL/6 J animals were intragastrically administered with distilled water or EEP for a treatment duration of 30 days. Upon sacrifice, careful scrutiny of the harvested mouse stomach tissues revealed a significant regression of the gross mucosal elevation in the *A4gnt* KO + EEP treatment group in comparison to the untreated control group (Fig. 2A, Supplementary Fig. 2) as essentially affirmed by semi-quantitative scoring analysis (Fig. 2D). Microscopically, this coincided with a sizeable reduction of the mean pyloric thickness in the EEP-treated *A4gnt* KO group with ~30% decrement as opposed to that of the corresponding control group given only distilled water (529.93 ± 45.08 μm vs 792.99 ± 105.90 μm) (Fig. 2B,E). In addition, subsequent examination of the CD3-positive T-lymphocytic cell infiltration in the *A4gnt* KO + EEP treatment group likewise depicted a marked decline in the mean cell count tallying only 20.59 ± 4.08 cells as compared to around 40.68 ± 12.94 cells obtained in the untreated control counterpart (Fig. 2C,F). Another thing worth mentioning also was the observation that no glaring difference could be deciphered between the distilled water and EEP-treated wildtype C57BL/6 J animals with respect to their gross morphology (Fig. 2A), gastric mucosal thickness (Fig. 2B), and T-lymphocyte sequestration (Fig. 2C). Therefore, on the basis of all these findings, it can be deduced that EEP may exert promising *in vivo* anti-tumor efficacy against differentiated-type gastric adenocarcinoma.

### EEP consistently affects cell cycle process *in vivo* to confer efficient anti-tumor action

**Figure 3:**
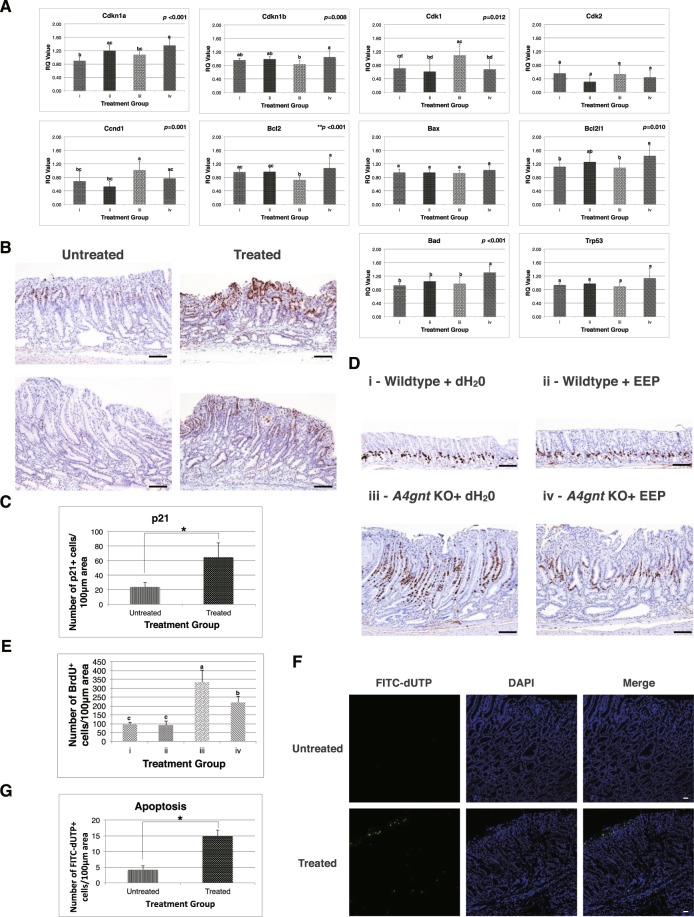
EEP consistently affects cell cycle process *in vivo* to confer efficient anti-tumor action. (**A**) mRNA expression levels of several genes related to cell cycle regulation and apoptosis among the different treatment groups using real time PCR. Data are shown as an average of two independent experiments with each analysis run in duplicates. Means with different letter are significant at *P* < 0.05 using ANOVA with Tukey-HSD posttest. ^**^P < 0.05 using Kruskal-Wallis test. (**B**) Representative sections of gastric pyloric mucosa depicting p21 immunoreaction between untreated and EEP-treated *A4gnt* KO mice. Scale bar: 100 μm (**C**) Comparison of the number of p21-positive cells between untreated and EEP-treated A4gnt KO animals expressed as mean counts per 100 μm taken from at least three different areas with the highest cell density. ^*^P < 0.05 using Independent sample t-test. (**D**) Representative sections of gastric pyloric mucosa among the different treatment groups showing BrdU labeling of actively dividing cells in the synthesis (S) phase of the cell cycle. Scale bar: 100 μm (**E**) Comparison of the number of BrdU-positive cells among the different treatment groups expressed as mean counts per 100 μm area taken from at least three different points with the highest cell density. Means with different letter are significant at *P* < 0.05 using ANOVA with Tukey-HSD posttest. (**F**) Representative sections of pyloric mucosa demonstrating cells undergoing apoptosis between untreated and EEP-treated *A4gnt* KO mice as revealed by TUNEL assay. Scale bar: 100 μm (**G**) Comparison of the number of FITC-dUTP-positive cells expressed as mean counts per 100 μm area taken from at least three different points with the highest cell density. *P* < 0.05 using Independent sample t-test. i – Wildtype + dH_2_0, ii – Wildtype + EEP, iii – *A4gnt* KO + dH_2_0, iv - *A4gnt* KO + EEP.

We next endeavored to search for specific mechanisms to which we could ascribe the pronounced *in vivo* anti-tumor action of our present EEP sample. In this connection, real time PCR technique was initially employed to inspect several inflammation-associated genes that are deemed crucial in *A4gnt* KO-induced carcinogenesis^28^. Upon gene expression analysis however, no overt changes were determined between the mRNA expression profiles of these genes in both *A4gnt* KO treatment groups except for a tendency for higher *Il10* transcription (Supplementary Fig. 3, Supplementary Table 3). Therefore, in light of this obvious indication for alternative mechanistic pathway, and in conjunction with the present *in vitro* data, we examined thereafter those genes associated with cell cycle progression and apoptosis. As illustrated in Fig. 3A and Supplementary Table 4, a specific and profound modulation was visibly observed in a number of cell cycle protein-encoding genes following EEP administration. In particular, *Cdkn1a* was considerably overexpressed (*p* < 0.001) in *A4gnt* KO + EEP treatment group and this notable increment as validated at the protein level (Figs. 3B,C) was documented to occur in a statistically different manner in relation to those of the untreated wildtype and *A4gnt* KO control animals. Similarly, this significant gene level augmentation was also found in the wildtype + EEP treatment group in contrast to the untreated wildtype counterpart thereby strongly supporting the gene-specific regulatory influence of EEP. In addition, *A4gnt* KO mice treated with EEP also exemplified a substantial increase in *Cdkn1b* (*p* < 0.008) and a decrease in *Cdk1* (*p* < 0.045) expressions in converse with the *A4gnt* KO + dH_2_O group. On a different note, assessment of the apoptosis-related genes between *A4gnt* KO treatment groups unveiled a perplexing interplay of several contrasting genes (*Bl2l1*, *Bcl2*, and *Bad*) (Fig. 3A, Supplementary Table 4), possibly insinuating the trivial relevance of this process in the perceived *in vivo* response.

To further contend that cell cycle participation constitutes a distinctive anti-cancer feature of our EEP sample, we then performed *in vivo* BrdU labeling. Interestingly, EEP supplementation in *A4gnt* KO mice elicited a remarkable diminution in the number of actively dividing BrdU-positive S-phase cells approximating only ~65% of the mean cell count registered in those of the corresponding KO control animals (220.45 ± 26.87 vs. 334.57 ± 59.08) (Fig. 3D,E). Meanwhile, BrdU labeling between wildtype treatment groups did not seem to solicit any statistical significance. Lastly, we aimed to validate the role of apoptosis by analyzing DNA fragmentation through TUNEL Assay. As demonstrated in Fig. 3F,G, the marginal increase in the number of cells undergoing apoptosis in the *A4gnt* KO + EEP treatment group as distinguished from its untreated counterpart, somehow reinforced the notion that this mechanism might only be accountable to a lesser extent in the *in vivo* anti-tumor action of the present EEP sample.

### Chemical composition of crude EEP

Preliminary phytochemical screening of the crude EEP from Philippine stingless bee yielded more than 500 chemical constituents encompassing widely diverse groups, which include carbohydrates, steroids, alkaloids, anthraquinones, phenols, terpenoids, etc. Of these, about 15 chemical compounds were identified as promising candidates with anti-cancer activity as supported by structure analysis and review of prior literature reports (Supplementary Table 5).

## Discussion

The bulk of the so-called “Pacific-type propolis” found in countries in the Far East region like Japan, Indonesia, Thailand, Taiwan, and Philippines is primarily produced by a distinct species of bees known as stingless bees. This propolis, as discriminated from those of the Brazilian green-type and Poplar-type, is exceptionally enriched in geranyl flavanone constituents instead of the commonly documented artepillin C or caffeic acid phenethyl ester (CAPE)^35^. Nevertheless, irrespective of the type, they are virtually comparable vis-à-vis the extent of their biofunctional activities such as anticancer, antimicrobial, anti-inflammatory, antioxidant, and wound healing properties^15,36–38^. In the Philippines, *Tetragonula biroi* Friese (syn *Trigona biroi*), locally recognized as “Kiwot”, comprise an important indigenous population of stingless bees. They are greatly valued as an effective pollinator and are mostly preferred for propolis production due to higher cost efficiency and increased resistance to parasitic infestation^39^. Expectedly, this has raised consciousness to strengthen studies on the multifaceted aspects of this bee propolis^27,40–42^, albeit, scientific inquiries relating to their biofunctional properties especially anti-tumor efficacy are lagging behind and remain nearly unstudied. Therefore, the present paper was designed to cater the apparent paucity of knowledge on the tumor-suppressing potential of Philippine stingless bee propolis. Here, we described that the EEP-induced reduction in gastric cancer cell proliferation *in vitro* and tumor growth regression *in vivo* were intimately associated with the specific and profound modulation of the components of the cell cycle machinery and in part, the apoptotic process.

Resistance against cancer growth and proliferation does no longer represent an unprecedented role for honeybee- and stingless bee-derived propolis. Accumulating lines of compelling evidence in literature have explicitly illuminated the causal relationship between propolis exposure and proliferative restriction in a broad range of human and murine *in vitro* cancer cell lines. For instance, propolis samples from the Thai stingless bees, *Trigona laeviceps*, and Indonesian stingless bees, *Trigona incisa, Timia apicalis, Trigona fusco-balteata*, and *Trigona fuscibasis* have exhibited marked cytotoxicity and anti-proliferative activity toward SW620 colon carcinoma, BT474 breast carcinoma, HepG2 hepatocellular carcinoma, and ChaGo-I lung carcinoma^43,44^ whereas Brazilian geopropolis from *Melipona scutellaris* stingless bees has been effective against U251 glioma, UACC-62 melanoma, MCF-7 breast adenocarcinoma, NCI-ADR/RES multi-drug resistant ovarian carcinoma, 786–0 renal carcinoma, NCI-H460 lung non-small cell carcinoma, PC-3 prostate carcinoma, and OVCAR-03 ovarian carcinoma^45^. In GC, strong evidence supporting this role comes from investigations using AGS, Kato III, NCI-N87, NUGC-4, MKN-1, and MKN-28 cells^43,46–48^. In the present paper, we further extended this growing list of tested GC cell lines by including MKN-45 and MKN-74 in addition to AGS and NUGC-4. These cells were preliminary classified into differentiated and diffuse subtype following the two-marker system of genomic GC classification by Tan and colleagues (2011)^31^. Based on the differential gene expression of adhesion molecules, *LGALS4* and *CDH17*, AGS, NUGC-4, and MKN-45 cells were categorized into the former while MKN-74 was designated into the latter. As demonstrated in this report, all the studied GC cell lines, except MKN-74, disclosed a concentration- and time course-dependent sensitivity to EEP exposure, although the generated IC_50_ value at 72 hours was relatively higher than those of earlier reported studies^46,48^. Nonetheless, we were able to document in this work that EEP from the Philippine stingless bees also possess promising anti-proliferative activity. More importantly, we have herein established the first account that EEP treatment may display GC subtype specificity showing prominent suppressive efficacy against the differentiated-type. However, one caveat of this finding was that the enhanced anti-proliferative activity might be due to cell line growth capacity^17^.

To provide a more meaningful assertion that EEP treatment could foster an increased differentiated GC subtype susceptibility, we utilized a spontaneous disease animal model of GC (*A4gnt* KO mice) that uniquely recapitulates a well-developed differentiated-type gastric adenocarcinoma at around 60-weeks of age. Upon oral supplementation of distilled water or EEP for 30 consecutive days, stomach tissue samples of EEP-treated *A4gnt* KO mice exemplified a remarkable regression of gross mucosal elevation, which corresponded histologically to substantial reduction of pyloric mucosal thickness and T-lymphocyte infiltration. These results strongly suggest that EEP treatment presumably mediates a significant tumor-demoting action against differentiated GC subtype. Congruent with these findings, oral administration of Iranian propolis significantly retarded the growth of gastric tumor lesions in Wistar rats following MNNG-initiated gastric carcinogenesis^49^. In another study using xenograft model of mammary carcinoma, water-soluble derivative of Croatian propolis successfully elicited an appreciably delayed tumor formation^50^. Meanwhile, hydroalcoholic extract of Brazilian green propolis evidently dampened the tumor growth induction in DMBA-induced mice model of dermal carcinogenesis although failed to impinge on inflammatory lymphocyte sequestration^51^. However, propolis treatment in all these above-mentioned studies, in converse to our study, has been carried out either prophylactically or concurrently with tumor induction. Therefore, the deliberate application of EEP under the circumstance of already existing tumor as in the case of *A4gnt* KO mice in this present work proffered an indisputable justification for its pronounced therapeutic anti-cancer property.

Cell cycle progression is a well-orchestrated process requiring tight coordination of the homeostatic balance between cyclin/cyclin-dependent kinase protein (CDK) complexes and their inhibitor proteins (CKI)^52^. Any perturbations involving its regulation have afforded cancers with unconstrained development^53,54^. Conceivably, targeted control of this mechanism has been inexorably exploited by most bioactive compounds and chemotherapeutic drug preparations. In this paper, we demonstrated the regulatory potential of our EEP sample on this process unveiling the exclusive and profound modulation of several cell cycle related gene transcripts in all differentiated-type GC cell lines. Specifically, *CDKN1A*, which encodes for the p21 protein, was strikingly upregulated, and this was accompanied by marked down-regulated levels of *CCND1*, *CDK1*, and *CDK2* genes encoding for the Cyclin D1, Cdk1, and Cdk2 proteins, respectively. Reconcilably, EEP treatment in *A4gnt* KO mice also led to a significant increase in the gene and protein levels of *Cdkn1a* coupled with a propensity to restore *Ccnd1* and *Cdk1* gene expressions. Moreover, this notable transcriptional modulation coincided with the prominent cell cycle cessation at the G0/G1 phase in AGS cells along with a marked reduction in the number of *in vivo* BrdU-positive S-phase cells in the *A4gnt* KO + EEP treatment group in comparison to the untreated KO control group. In consonance with our data, exposure to propolis and its bioactive components has stimulated a significant increment in p21 mRNA and protein expressions resulting to cell cycle arrest at G0/G1 phase of various human cancer cell lines of colon and lung carcinoma^56–58^. Intriguingly, this increase in G1 cells as validated using the bi-parametric BrdU/DNA cell cycle analysis in another study essentially correlated to a reduced S-phase cell population^33^. p21, also known as p21^WAF1, CIP1, SDI1, CAP20, MDA6^, belongs to the Cip/Kip family of cyclin-CDK complex inhibitors that play diverse roles including cell cycle control, transcriptional regulation, senescence, cell differentiation, cytoskeletal dynamics, and apoptosis^55^. It can mediate a p53-dependent G1 growth arrest through inhibition of cyclin D/Cdk4 and Cyclin E/Cdk2 complexes following DNA damage and oxidative stress^59,60^. Alternatively, in a p53-independent manner, it may also serve as a master growth suppressing effector by blocking G1/S transition via disruption of Cyclin E/Cdk2 and Cyclin A/Cdk2 complexes thus promoting phosphorylation of retinoblastoma (RB) and sequestration of E2F1; as well as regulating G2/M checkpoint through repressive action on Cyclin B1/Cdk1 and Cyclin A/Cdk1^55^. Therefore, drawing from these accounts, it seems reasonable to imply that p21-induced G0/G1 phase arrest mediates the significant tumor suppressing potential of our present EEP sample.

We cannot also exclude the probability of the apoptotic machinery assisting synergistically in the anti-tumor efficacy of our EEP sample. In the current work, although we did not find any distinct consensus regarding the gene transcriptional profile of selected apoptosis markers in differentiated-type GC cell lines after EEP treatment, these cells manifested a response indicative of such phenomenon including the significant overexpression of pro-apoptotic genes, *BAX* and *BAD* in AGS cells along with the down-regulation of the anti-apoptotic genes, *BCL2L1* in MKN-45 and *BCL2* in NUGC-4 cells. As verified through TUNEL assay, the disparate degrees of DNA fragmentation occurring in these cell lines may potentially explain this apparent disaccord in gene expression. Concordantly, propolis administration has similarly provoked the differential induction of apoptosis-associated markers in previous studies of various human cancer cell lines. While it has initiated DNA fragmentation via activation of *BAX* and inhibition of *BCL2* and *BCL-XL* (encoded by the *BCL2L1* gene) in laryngeal carcinoma Hep-2 cells and colon carcinoma HL-60 cells^33,61^, a decrease in *BCL2* without alteration in *BAX* was reported in another study using U937 leukemic cells^24^. At present, we could hardly offer any substantial rationalization to what seems to be a specific regulatory role of our propolis sample on the *in vivo* gene expressions of *Bad* and *Bcl2l1* in expending its apoptotic effect. Whether this simultaneous induction indicates a compensatory response remains to be elucidated. Nevertheless, the significant augmentation of both of these genes in *A4gnt* KO + EEP treatment group that was reminiscent of those of AGS cells, the absence of substantial sub-G1 phase accumulation in AGS cells, and the marginal *in vivo* induction of FITC-dUTP-positive apoptotic cells likely suggest the trifling involvement of this mechanism in the tumor-suppressing potential of our present EEP sample. Additionally, the formerly held perception that an active cell cycle was a requisite for apoptosis induction and that p21 stimulation conferred protection against apoptosis by cell cycle disruption^55^ further supplemented this contention.

Finally, we sought to tentatively identify some candidate compounds in our crude EEP sample that may possibly serve as useful chemical markers with anti-cancer efficacy. Since few chemical-profiling studies by LC-MS analysis have already been conducted on samples of Philippine stingless bee propolis including those obtained from the same colonies as in the present work^41,42^, we decided to utilize a different analytical platform, hence employing GC-MS/MS analysis. Out of over 500 chemical components herein identified, 15 were preliminarily selected as potential candidate compounds mostly belonging to the terpenoid and phenolic acid groups. For example, β-eudesmol and guaiol are both classified under the sesquiterpene class of terpenoids. These compounds have been commonly reported as major constituents of propolis samples from Lebanon, Turkey, Croatia, Greece, China, and Brazil^62–67^. Interestingly, both have been well established to mediate anticancer activity either through G1 phase arrest and caspase-initiated apoptosis^68^ or through mTOR signaling pathway control^69^. On the other hand, gallic acid, protocatechuic acid, and pterostilbene were some of the identified phenolic acids. The former two compounds have been previously detected in Chinese, Uruguayan, Brazilian, Greek, and Cypriot propolis^70,71^ whereas the latter has been found almost exclusively in samples of Australian propolis^13^. Treatment with gallic acid and pterostilbene has been earlier shown to elicit significant growth restriction of MDA-MB-231 triple-negative breast adenocarcinoma and AGS gastric carcinoma^72,73^. This marked inhibition necessitated in part the modification of the cell cycle process via p21- and p27- induced G1 phase blockage, which is compatible with our current findings.

Further testing of the these selected candidate compounds, whether alone or in combination, on different human cancer cell lines as well as in *in vivo* cancer models like *A4gnt* KO mice may provide a more meaningful validation of their applicability as a potential anticancer therapeutic agent with cytostatic or cytotoxic properties.

## Supplementary information

Supporting information

## Data Availability

All datasets analyzed and/or generated during the conduct of this present work are available from the corresponding author upon reasonable request.
