# Supplementary material for: Tumor-suppressing potential of stingless bee propolis in in vitro and in vivo models of differentiated-type gastric adenocarcinoma
Source: Sci Rep. 2019 Dec 23;9:19635. doi: 10.1038/s41598-019-55465-4 (PMC6928070; doi:10.1038/s41598-019-55465-4)
Supplement: Supplementary file 1 — Supporting information [file 41598_2019_55465_MOESM1_ESM.pdf]

Supplementary Information

Tumor-suppressing potential of stingless bee propolis in preclinical models of intestinal-type gastric adenocarcinoma

Mark Joseph Desamero[1, 2, 3], Shigeru Kakuta[1*],Yulan Tang[1], James Kenn Chambers[5], Kazuyuki Uchida[5], Maria Amelita Estacio[2, 3], Cleofas Cervancia[3, 4], Yuri Kominami[6], Hideki Ushio[6], Jun Nakayama[7], Hiroyuki Nakayama[5], Shigeru Kyuwa[1]

[1]Department of Biomedical Science, Graduate School of Agricultural and Life Sciences, The University of Tokyo, 1-1-1, Yayoi, Bunkyo-ku, Tokyo, 113-8657, Japan

[2]Department of Basic Veterinary Sciences, College of Veterinary Medicine, University of the Philippines Los Baños, Laguna, 4031, Philippines

[3]UPLB Bee Program, University of the Philippines Los Baños, Laguna, 4031, Philippines

[4]Institute of Biological Sciences, College of Arts and Sciences, University of the Philippines Los Baños, Laguna, 4031, Philippines

[5]Department of Veterinary Pathology, Graduate School of Agricultural and Life Sciences, The University of Tokyo, 1-1-1, Yayoi, Bunkyo-ku, Tokyo, 113-8657, Japan

[6]Laboratory of Marine Biochemistry, Graduate School of Agricultural and Life Sciences, The University of Tokyo, 1-1-1, Yayoi, Bunkyo-ku, Tokyo, 113-8657, Japan

[7]Department of Molecular Pathology, Graduate School of Medicine, Shinshu University, 3-1-1 Asahi, Matsumoto, Nagano, 3908621, Japan

*Corresponding Author: akakuta@mail.ecc.u-tokyo.ac.jp, Department of Biomedical Science, Graduate School of Agricultural and Life Sciences, The University of Tokyo, 1-1-1, Yayoi, Bunkyo-ku, Tokyo, 113-8657, Japan

SUPPLEMENTARY FIGURES

Supplementary Figure 1. mRNA expression levels of adhesion molecules, *CDH17* and *LGALS4* in four human GC cell lines.

	AGS	MKN-45	NUGC-4	MKN-74
CDH17	1.00	2.56	-	0.01

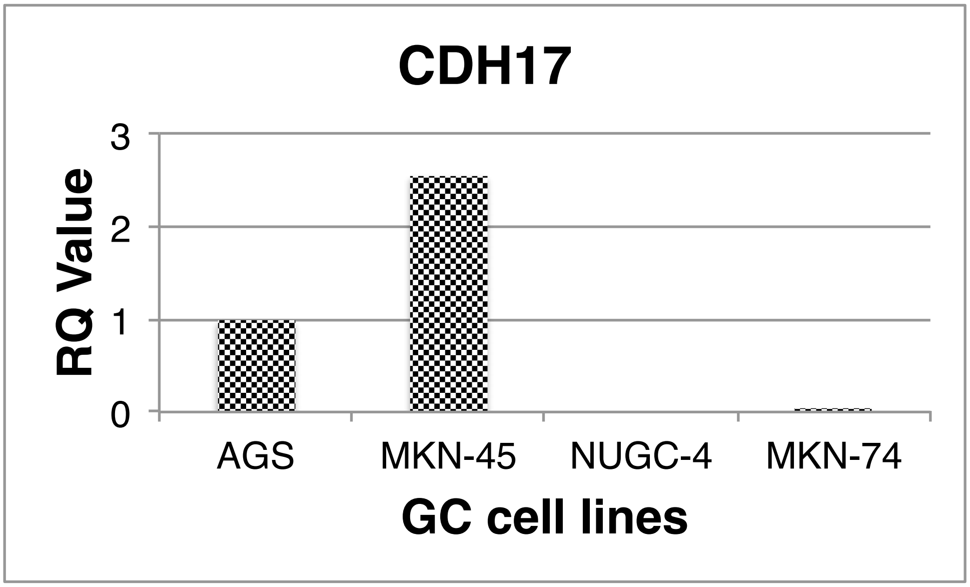

	AGS	MKN-45	NUGC-4	MKN-74
LGALS4	1.00	66.27	89.01	0.06

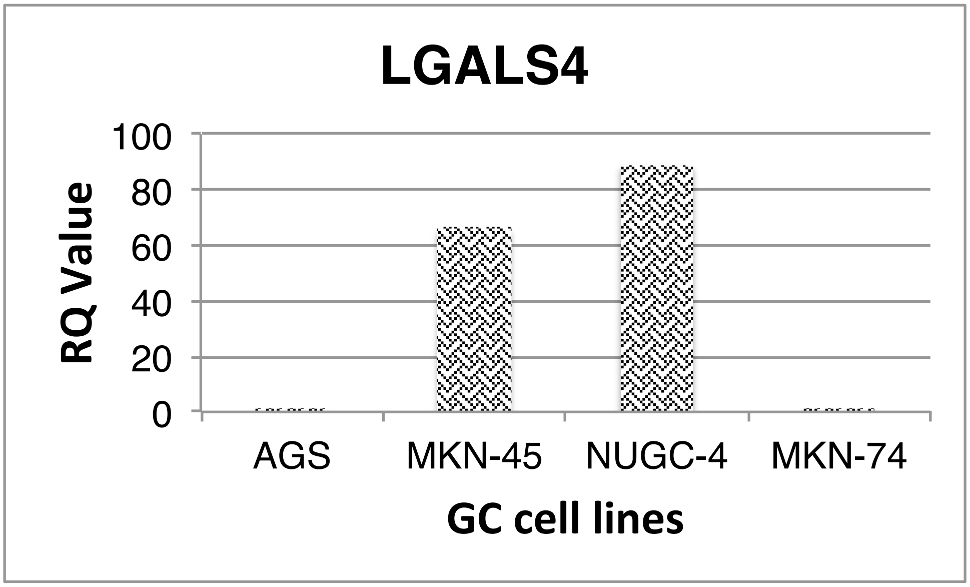

Supplementary Figure 2. Gross morphology of the harvested mouse stomach tissues in four different treatment groups.

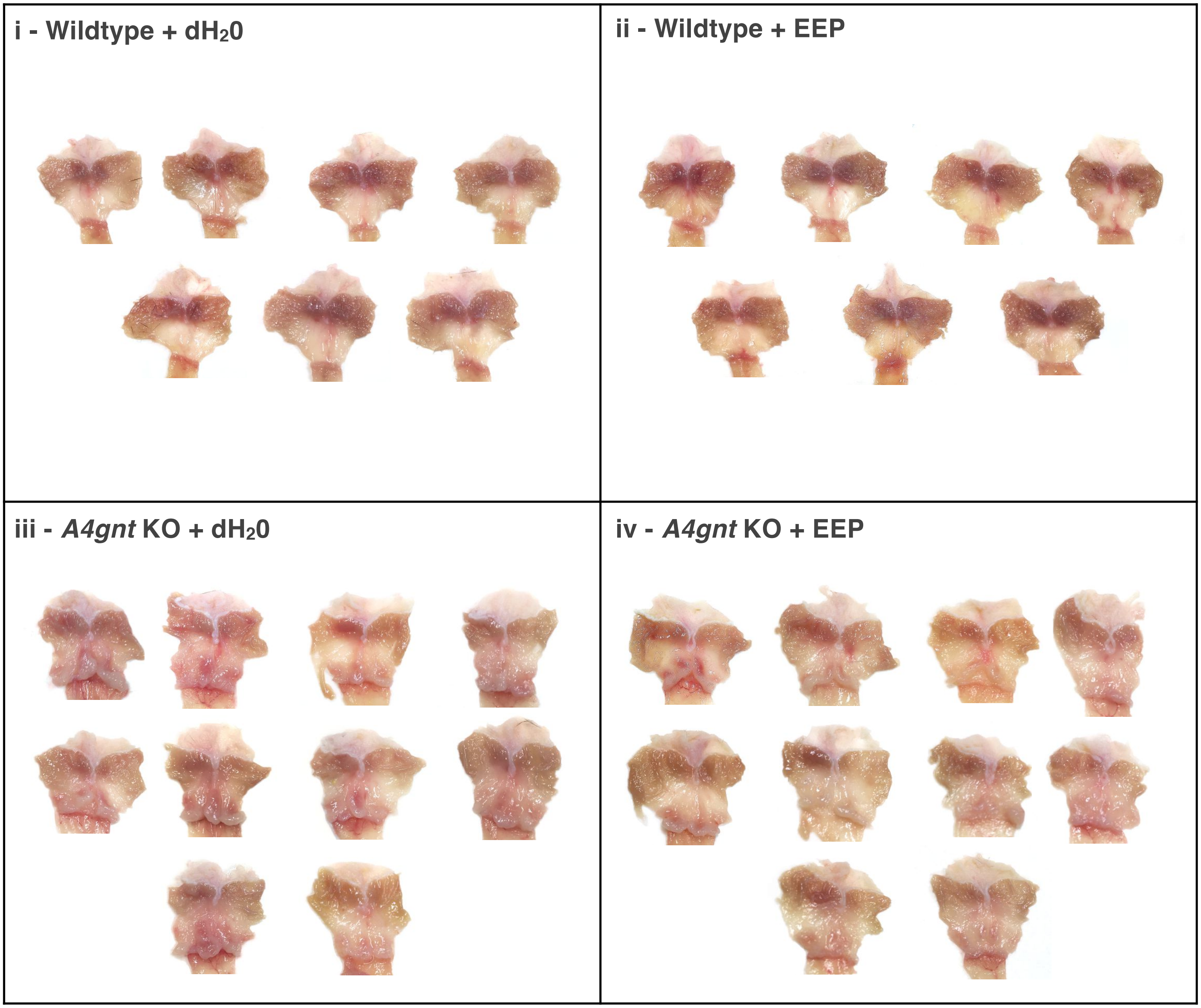

Supplementary Figure 3. mRNA expression levels of selected inflammation-related genes in three different treatment groups.

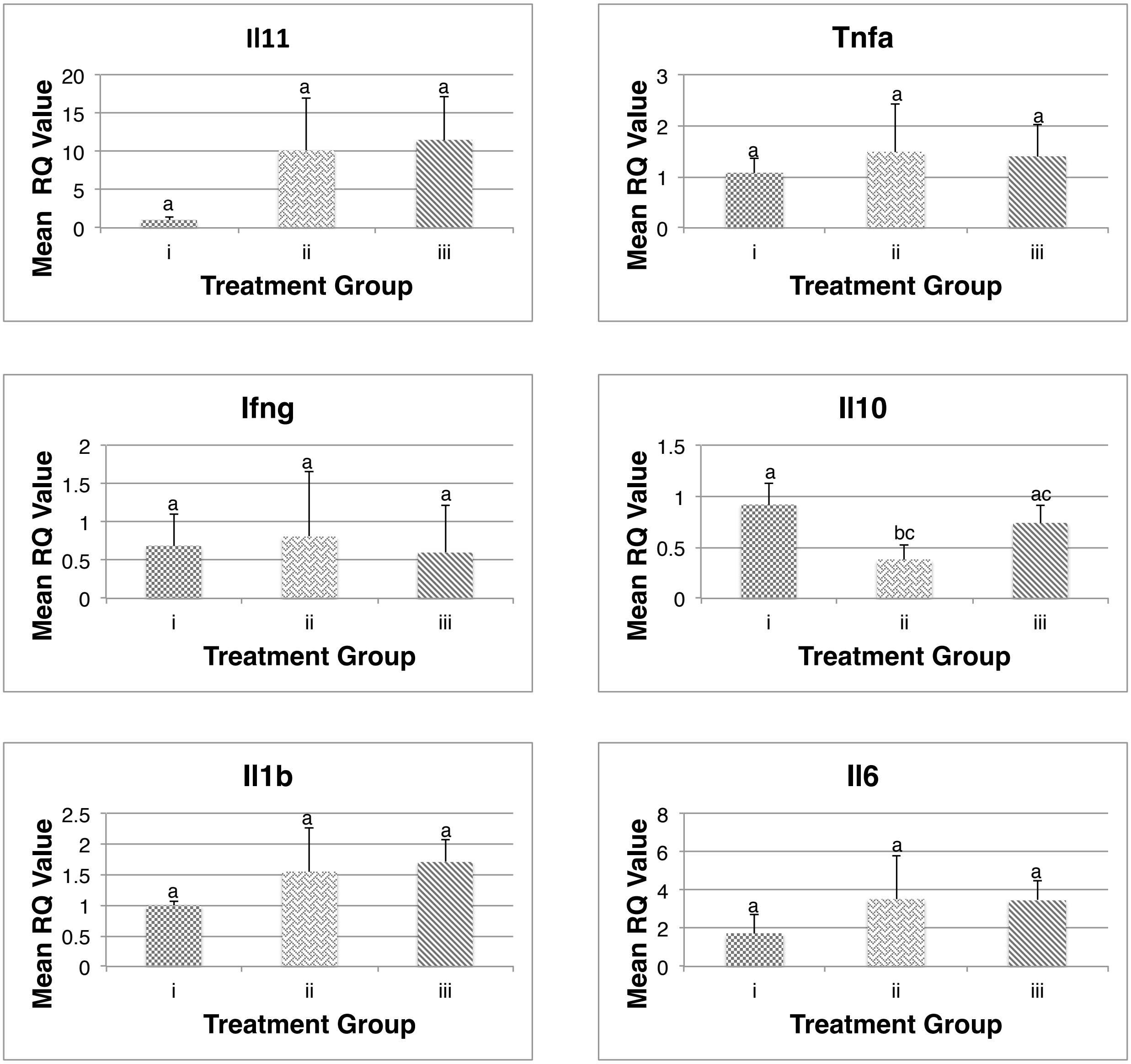

SUPPLEMENTARY TABLES

Supplementary Table 1. Primer details of selected genes utilized in the present study.

	Genes	Forward	Reverse
**Mouse**	*Actb*	AAGTGTGACGTTGACATCCG	GATCCACATCTGCTGGAAGG
	Il10	GCTCTTACTGACTGGCATGAG	CGCAGCTCTAGGAGCATGTG
	Il11	TGTTCTCCTAACCCGATCCCT	CAGGAAGCTGCAAAGATCCCA
	Il1b	GCAACTGTTCCTGAACTCAACT	ATCTTTTGGGGTCCGTCAACT
	Tnfa	CCCTCACACTCAGATCATCTTCT	GCTACGACGTGGGCTACAG
	Ifng	ATGAACGCTACACACTGCATC	CCATCCTTTTGCCAGTTCCTC
	Il6	TAGTCCTTCCTACCCCAATTTCC	TTGGTCCTTAGCCACTCCTTC
	Bcl2	ATGCCTTTGTGGAACTATATGGC	GGTATGCACCCAGAGTGATGC
	Bcl2l1	GACAAGGAGATGCAGGTATTGG	TCCCGTAGAGATCCACAAAAGT
	Bax	TGAAGACAGGGGCCTTTTTG	AATTCGCCGGAGACACTCG
	Bad	AAGTCCGATCCCGGAATCC	GCTCACTCGGCTCAAACTCT
	Trp53	GCGTAAACGCTTCGAGATGTT	TTTTTATGGCGGGAAGTAGACTG
	Cdkn1a	CCTGGTGATGTCCGACCTG	CCATGAGCGCATCGCAATC
	Cdkn1b	TCAAACGTGAGAGTGTCTAACG	CCGGGCCGAAGAGATTTCTG
	Cdk1	AGAAGGTACTTACGGTGTGGT	GAGAGATTTCCCGAATTGCAGT
	Cdk2	CCTGCTTATCAATGCAGAGGG	GTGCTGGGTACACACTAGGTG
**Human**	*ACTB*	AAGTGTGACGTTGACATCCG	GATCCACATCTGCTGGAAGG
	BCL2	GGTGGGGTCATGTGTGTGG	CGGTTCAGGTACTCAGTCATCC
	BCL2L1	GACTGAATCGGAGATGGAGACC	GCAGTTCAAACTCGTCGCCT
	BAX	CCCGAGAGGTCTTTTTCCGAG	CCAGCCCATGATGGTTCTGAT
	BAD	CCCAGAGTTTGAGCCGAGTG	CCCATCCCTTCGTCGTCCT
	TP53	CAGCACATGACGGAGGTTGT	TCATCCAAATACTCCACACGC
	CDKN1A	TGTCCGTCAGAACCCATGC	AAAGTCGAAGTTCCATCGCTC
	CDKN1B	AACGTGCGAGTGTCTAACGG	CCCTCTAGGGGTTTGTGATTCT
	CCND1	GCTGCGAAGTGGAAACCATC	CCTCCTTCTGCACACATTTGAA
	CDK1	AAACTACAGGTCAAGTGGTAGCC	TCCTGCATAAGCACATCCTGA
	CDK2	CCAGGAGTTACTTCTATGCCTGA	TTCATCCAGGGGAGGTACAAC

Supplementary Table 2. Summary data of the mean mRNA expression levels of selected cell cycle- and apoptosis related genes in four human GC cell lines.

	**AGS**			**MKN-45**			**NUGC-4**			**MKN-74**		
	Untreated	Treated	*p* value	Untreated	Treated	*p* value	Untreated	Treated	*p* value	Untreated	Treated	*p* value
CDKN1A	1.23±0.21	13.18±1.20	< 0.001	1.01±0.45	3.62±3.79	0.302	0.91±0.09	4.80±1.10	* 0.050	1.05±0.13	0.92±0.13	0.300
CDKN1B	1.15±0.16	2.38±0.17	0.001	0.81±0.22	0.43±0.11	0.057	1.16±0.15	0.98±0.18	0.248	1.07±0.06	0.55±0.11	*0.002
CDK1	0.90±0.09	0.11±0.01	* 0.050	1.06±0.06	0.19±0.01	< 0.001	1.05±0.13	0.16±0.04	< 0.001	1.05±0.10	0.57±0.02	** 0.050
CDK2	0.89±0.10	1.04±0.05	0.077	1.07±0.17	0.53±0.05	0.006	0.91±0.20	0.15±0.03	* 0.050	1.07±0.11	0.95±0.21	0.438
CCND1	1.08±0.08	1.89±0.06	< 0.001	1.42±0.66	0.46±0.01	* 0.050	1.05±0.11	0.39±0.12	0.002	0.99±0.03	0.54±0.02	*< 0.001
BCL2	1.11±0.26	0.66±0.17	0.068	0.74±0.30	0.27±0.21	0.084	0.97±0.03	0.07±0.03	< 0.001	1.16±0.17	1.53±0.40	0.210
BCL2L1	0.84±0.14	3.40±0.40	< 0.001	1.00±0.08	0.44±0.04	* 0.050	0.96±0.06	1.01±0.18	0.062	0.99±0.12	0.36±0.07	*0.002
TP53	0.99±0.04	1.35±0.10	0.005	0.98±0.12	0.71±0.06	0.241	1.02±0.05	0.78±0.11	0.027	0.93±0.06	0.51±0.10	*0.003
BAX	1.05±0.10	2.64±0.26	0.001	0.70±0.32	0.44±0.45	0.447	0.90±0.09	1.13±0.25	0.203	1.07±0.10	1.19±0.09	0.190
BAD	0.98±0.08	1.65±0.17	0.003	0.86±0.27	0.54±0.52	0.393	0.98±0.06	0.42±0.09	0.001	1.09±0.08	0.60±0.05	*0.001

* Independent Sample T-test at $p < 0.05$
** Mann-Whitney U test at $p < 0.05$

Supplementary Table 3. Summary data of the mean mRNA expression levels of selected inflammation-related genes in three different treatment groups.

		**Genes**					
		Il11	*Tnfa*	*Ifng*	*Il10*	*Il1b*	*Il6*
i	Wildtype + dH_20	1.03±0.48	1.08±0.29	0.69±0.41	0.92±0.21	1.00±0.07	1.72±1.00
ii	*A4gnt* KO + dH_20	10.15±6.88	1.49±0.94	0.81±0.86	0.38±0.14	1.55±0.73	3.53±2.26
iii	*A4gnt* KO + 30% EEP	11.49±5.64	1.40±0.62	0.60±0.61	0.74±0.17	1.71±0.36	3.46±1.01
	p value	**0.055	0.727	0.915	*0.02	**0.055	0.285

* ANOVA with Tukey-HSD posttest at $p < 0.05$
** Kruskal-Wallis Test at $p < 0.05$

Supplementary Table 4. Summary data of the mean mRNA expression levels of selected cell cycle- and apoptosis related genes in four different treatment groups.

	Treatment Groups				
	i	ii	iii	iv	
Genes	Wildtype + dH_20	Wildtype + 30% EEP	*A4gnt* KO + dH_20	*A4gnt* KO + 30% EEP	*p* value
Cdkn1a	0.90±0.11	1.20±0.18	1.08±0.12	1.36±0.19	*< 0.001
Cdkn1b	0.96±0.06	0.99±0.09	0.84±0.11	1.05±0.21	*0.008
Cdk1	0.71±0.32	0.61±0.37	1.10±0.33	0.68±0.32	*0.012
Cdk2	0.56±0.29	0.31±0.18	0.54±0.25	0.44±0.21	0.088
Ccnd1	0.69±0.29	0.53±0.16	1.02±0.26	0.77±0.23	*0.001
Bcl2	0.96±0.09	0.97±0.12	0.73±0.10	1.08±0.30	**< 0.001
Bcl2l1	1.12±0.18	1.26±0.25	1.09±0.24	1.44±0.30	*0.010
Trp53	0.94±0.06	0.98±0.06	0.90±0.11	1.14±0.29	0.076
Bax	0.95±0.10	0.95±0.08	0.93±0.09	1.02±0.13	0.225
Bad	0.93±0.07	1.05±0.13	0.98±0.19	1.31±0.19	*< 0.001

* ANOVA with Tukey-HSD posttest at $p < 0.05$

** Kruskal-Wallis Test at $p < 0.05$

Supplementary Table 5. Selected compounds obtained from the crude extract of Philippine stingless bee propolis.

Pubchem CID	Compound	Molecular Formula	MW
227829	Guaiol	$C_{15}H_{26}O$	222
444008	Tibolone	$C_{21}H_{28}O_2$	312
5318517	Andrographolide	$C_{20}H_{30}O_5$	350
370	Gallic acid	$C_7H_6O_5$	170
91457	β-Eudesmol	$C_{15}H_{26}O$	222
2950	Danthron	$C_{14}H_8O_4$	240
6324617	Ginkgolide-B	$C_{20}H_{24}O_{10}$	424
444539	Cinnamic acid	$C_9H_8O_2$	148
6167	Colchicine	$C_{22}H_{25}NO_6$	399
528594	Protocatechuic acid	$C_{25}H_{48}O_4Si_3$	496
5281858	Ginkgolic acid	$C_{22}H_{34}O_3$	346
5281251	Rhodoxanthin	$C_{40}H_{50}O_2$	562
5281727	Pterostilbene	$C_{16}H_{16}O_3$	256
13966122	Rosmanol	$C_{20}H_{26}O_5$	346
31404	Butylated hydroxytoluene	$C_{15}H_{24}O$	220
